# How General Is the Semantic Structure of Time? A Comparison of Indians and Germans

**DOI:** 10.1007/s12124-020-09520-9

**Published:** 2020-03-19

**Authors:** Peter Sedlmeier, Babu Rangaiah, Doreen Weber, Isabell Winkler

**Affiliations:** 1 Department of Psychology, Chemnitz University of Technology, 09107 Chemnitz, Germany; 2 Pondicherry University, Pondicherry, India

**Keywords:** CUBR-D-19-00026, Semantic structure of time, Intercultural, Indians, Germans

## Abstract

People in different cultures differ in their time-related behaviors and judgments. But do they also differ in how time is represented in their minds, that is, in their semantic structures of time, and if so, how and why? Two studies addressed these questions using participants’ time-related associative responses to compare the semantic structures of time across Indian and German university students. Study 1 compared time-related associations and found only low intercultural agreement, which increased somewhat if associations were grouped into categories. In Study 2, a comparison of the results of multidimensional scaling analyses on a cross-culturally representative selection of stimuli was consistent with the conclusion that differences across cultures are much more pronounced than commonalities. Two cultural aspects in particular might be responsible for the diversity in the semantic structures of time: the monochronic–polychronic distinction and the distinction between linear and cyclical time. Moreover, intercultural differences may be strongly intensified by language effects, especially if the languages in question greatly differ. It is concluded that behavioral and judgmental differences in dealing with time may be grounded in how people intuitively think about it and the language used to do so.

Time is an elusive concept, as expressed by Saint Augustine^1^ in his *Confessions* (Book XI)^1^: “What then is time? If no one asks me, I know what it is. If I wish to explain it to him who asks, I do not know.” This may be a familiar experience to many if not all of us. We all have a semantic structure of time but it is hard to express what exactly this often largely implicit structure is. If direct methods to elicit this semantic structure of time do not work well, it might be a promising idea to use indirect ones instead. This is the governing idea in the research presented here. Moreover, if one wants to find out how general such a semantic structure is, a good strategy might be to compare people from markedly different cultural contexts and explore whether and how they differ. In respect to the semantic structure of time, a comparison of western and eastern cultures appears to be especially promising. There seems to be widespread consensus that westerners differ from easterners in at least two aspects of their worldview that might have an impact on people’s semantic structure of time. As detailed below, these differences should be especially pronounced when comparing a western culture, such as that of Germany, to a specific eastern one: that of India.

## Two Time-Relevant Cultural Differences Between Indians and Germans

The first difference between westerners and easterners that might have an impact on people’s semantic structure of time concerns the distinction between monochronic and polychronic time use, introduced by Hall (1959; see also Hall and Hall 1987). Following monochronic time (M-time) means concentrating on only one thing at a time, whereas following polychronic time (P-time) means being involved with many things at once. M-time is almost tangible: Time can be “spent,” “saved,” “wasted,” and “lost.” M-time people do not like to be interrupted when working on a task, and they intensify some relationships while excluding others, for want of time. In contrast, P-time is full of interruptions and is characterized by great involvement with people; switching among activities is seen as desirable and productive, in the respective cultures. P-time people are more concerned with completing human transactions than with keeping schedules.^2^ According to Hall and Hall (1987, p. 17), time in Germany, as well as other northern European countries and the United States, is a classic example of an M-time system, whereas southern and eastern countries, such as India, tend to follow P-time systems.

The second aspect of time that differs between western and eastern cultures refers to the distinction between a linear and a cyclical notion of time (e.g., Toynbee 1956), the former connected to Semitic (Judaism, Christianity, and Islam) and the latter to Indic (Hinduism, Buddhism) religions. The usual and for westerner natural way to view time is that it progresses linearly, as suggested by clock time (e.g., Calkins 1970; Saunders et al. 2004; Zakay and Fleisig 2011), although several philosophers find this view problematic (Klempe 2015). There seems, however, general agreement that time is irreversible (e.g., Valsiner 1994, 2002). Especially the assumption about a linearly progressing time contrasts with the notion of cyclical time that in eastern cultures comes in two varieties (see Balslev 1999). One stems from ancient Indian Hindu systems of thought and refers to the assumption that “the cosmos passes through cycles within cycles for all eternity” (Basham 1956, p. 320). However, these cycles that describe the cosmological process as a never-ending series of repeated creations and dissolutions are conceived in terms of billions of human years (Balslev 1999, p. 146) and might not be of much consequence in Hindus’ daily life. Furthermore, there has been some discussion as to whether respective western descriptions of the Hindu notion of time as cyclical are really well founded (Sharma 1974). The second notion of cyclical time is a different case, one that depends heavily on the doctrine of *karma*. This law of cause and effect plays a central role in practically all the major ancient and contemporary Indian schools of thought (Neufeldt 1986; Phillips 2009), as well as in Indian daily life (Chaudhary 2015; Pattanaik 2003). It basically says that people create their *samsara*, that is, their cyclic wheel or chain of existence, or, in other words, their destiny, through their intentions and actions. Strongly connected with the notion of karma is that of reincarnation/transmigration (Hinduism) or rebirth (Buddhism): Human existence is going beyond birth and death.^3^ The cycle of birth and death can only be ended by reaching the goal of all major Indian thought systems, variously termed enlightenment, liberation, or salvation. This goal is reached when all bad karma is gone by striving for right or wholesome intentions and actions and avoiding unwholesome ones. However, unless people dedicate their life to the pursuit of a spiritual path, they can expect to still have many lives ahead. These repetitions of births and deaths cannot really be seen as exact replications but rather as a spiral moving toward the goal of liberation. Nonetheless, if there are many lives ahead, there is no pronounced need to feel time pressure because if the current life ends, there will be another one. This is substantially different from the conception of one and only one life, the linear conception of time, of which most westerners are convinced.

## Measuring the Semantic Structure of Time: An Associative Approach

Although seldom found in linguistics, there is a long tradition in psychology of exploring semantic structures, that is, internal representations that help people understand the world and themselves, by using associations between words (Brown and Berko 1960; Burgess 1998; Clark 1970; Landauer and Dumais 1997; Wettler et al. 2005). The details about doing so differ widely but the basic assumption is the same: Associative structures between words represent semantic structures. We use this assumption in exploring the semantic structure of time (for a thorough discussion of different assumptions about the linguistics of meaning see Cornejo 2004). Intercultural differences in associations between words may be heavily dependent on participants’ linguistic background, especially their mother tongues (e.g., Boroditsky 2001; Sedlmeier et al. 2016). Thus, it is desirable to separate the effects of cultural differences not connected to language, such as the two aspects of time discussed above, and effects that depend on language. One way to explore the additional impact of the linguistic background is to use a third language for comparing culturally diverse groups, with English often being the most suitable candidate because of its widespread use (e.g., Boroditsky et al. 2003).

If an associative structure for a given topic is to be modeled, one could use words from previous studies on that topic, as has been done by Pollio et al. (2014). Taking items on time from four published sources, and having their participants sort these items into categories, these authors found quite similar clustering patterns in two separate studies. This result could be indicative of a universal semantic structure of time, although conclusions are limited by the two samples having stemmed from the same population, American graduate students in psychology and education. We decided on a different procedure, for two reasons. First, preselected terms might not be representative of the terms participants are most familiar with, and second, even more important in our context, terms used in one culture might be understood differently in another culture, or not be used at all. Therefore, in a first study, we had participants in India and Germany produce their own time-related associations, a representative sample of which, in a second study, was used to make judgments about associative strengths. To minimize the variance due to a noncomparability of the samples in the two countries, only university students with a good command of English were included in the studies.

## Study 1: What Do People Think if they Think of Time?

In Study 1, we wanted to obtain evidence on whether participants’ time-related associations were comparable across different cultures and languages, or not. The former should be the case if there is a universal semantic structure of time, and the latter if the conception of time is highly context-dependent. Whereas in Germany, the vast majority of the population speak German as their mother tongue, the state of affairs in India is quite different. There, people speak a multitude of languages that stem from several distinct language families (e.g., Pattanayak 1990). For our study, we chose Kannada, a Dravidian language mostly spoken in the southern Indian state of Karnataka, because BR (the second author) is a native speaker of that language. Language is, of course, part of the culture but for people who speak several languages, different semantic structures might be activated when speaking different languages. Therefore, both in Germany and India, we took a sample that was to use their mother tongue and a sample that was to use a common second language: English.

### Methods

#### Participants

All participants were students of psychology or the social sciences. The two Indian samples were taken from Karnatak University, Dharwad (Kannada language: *n* = 27, mean age = 22.7 years, 69% female), and Bangalore University as well as Pondicherry University (English language: *n* = 51, mean age = 25.5 years, 33% female). The two German samples were both from the University of Technology, Chemnitz (German language: *n* = 38, mean age = 22.1 years, 79% female; English language: *n* = 38, mean age = 22.4 years, 66% female). All data were collected in classroom settings, and participants could obtain course credit for participation.

#### Task

Participants were handed a sheet of paper headed “Associations with TIME” with the following instructions (English version): “Please write down 20 English words, which you associate with ‘time.’” Below that, 20 numbered lines were provided on the sheet. In the Kannada and German versions, all instructions were given in the respective languages, and in the Kannada version, also the Kannada script was used. Both the Indian and German samples that completed the English version of the task were asked to rate their proficiency in English from 1 (*none*) to 10 (*fluent in English*). The mean ratings were quite comparable: 7.8 for the Indian, and 7.2 for the German sample.

### Results

In all four samples together, participants associated a total of 681 different words or expressions with “time.” The number of different words in the two Indian samples was *n* = 215 for the Kannada language sample and *n* = 388 for the English language sample.^4^ The respective numbers for the two German samples were *n* = 268 (German language) and *n* = 230 (English language). If there exists a uniform semantic structure of time, independent of culture and language, one would expect largely similar associations and therefore also largely equivalent numbers of associations for the total number of participants as well as for each of the four samples, especially for the largest one. This was definitely not the case. Thus, already these first numbers indicate that the four samples might differ in their associations and this might be partly dependent on the language used. However, many of these words or expressions were named by only one participant and therefore may not be representative of the respective culture.

#### How Well Do Specific Associations Agree?

Table 1 shows all associations produced by more than 10% of the participants in the respective samples. If one just reads the respective top column entries, the German samples exhibited associations that deal prominently with the measurement of time (e.g., clock, day, years, watch, second, hour, minute) whereas the Indian samples seem to have associatively connected “time” strongly to student life (e.g., working, examination, college, friends, class). Another difference to be noted in Table 1 is that the distributions of the ordered percentages are markedly more “skewed” for the German (maxima: 92.1% and 81.1%, for “clock” in both samples) than the Indian (maxima: 45.1% for “examination” and 55.6% for “working”) samples. Some associations were found only in the Indian (e.g., “prayer”) or the German (e.g., “stress”) lists. For the analysis, we considered only associative responses that were produced by more than 10% of the participants in the respective groups. Taking the respective smaller number of such associations in Table 1 as a basis,^5^ the amount of exact agreement in the associations across the two languages for the Indian samples is 51% (24 of 47); for the German samples, the respective figure is 61% (30 of 49). To measure the agreement across cultures, it seems most appropriate to assess the two samples using the English language. Here we obtain an associative agreement of only 26% (12 of 47). If we compare the samples that differ in both culture and language, the agreement is as low as 17% (10 of 58). Thus, the results of this first analysis indicate cultural differences in respect to the semantic structure of time between Indian and German students, as well as additional differences presumably depending on language.^6^ An indicator of the impact of language (probably a restricted possibility of expression in the foreign language) might be seen in the fact that the number of common associations produced by more than 10% of the respective participants was markedly lower in the groups that used English compared to the groups that used their native language (see Table 1).

**Table 1:** Rank-ordered percentages of participants associating words or expressions with “time” in each of the four samples (percentages smaller than 10% omitted)

Germany				India			
German (*n* = 38)	Percentage	English (*n* = 38)	Percentage	English (*n* = 51)	Percentage	Kannada (*n* = 27)	Percentage
Clock	92.1	Clock	81.6	Examination	45.1	Working	55.6
Second	50.0	Day	55.3	Friends	37.3	College	40.7
Hour	47.4	Years	52.6	Class	35.3	Examination	40.7
Minute	47.4	Watch	47.4	Working	35.3	Home	37.0
Stress	47.4	Minute	44.7	Watch	33.3	Sleeping	37.0
Day	39.5	Month	44.7	Important	31.4	TV	37.0
Years	31.6	Life	39.5	Sleeping	31.4	Valuable	37.0
Aged	28.9	Second	36.8	Valuable	29.4	Catch bus	33.3
Month	28.9	Fast	28.9	Precious	27.5	Class	33.3
Leisure	26.3	Late	28.9	Clock	25.5	Life	29.6
Life	26.3	Stress	28.9	Money	25.5	Friends	25.9
Appointment	23.7	Summer	28.9	Research	25.5	Important	25.9
Holiday	23.7	Future	26.3	Catch bus	23.5	Job	25.9
Alarm	21.1	Old age	26.3	Department	23.5	Meals	25.9
Boring	21.1	Aged	23.7	Life	23.5	Playing	25.9
Future	21.1	Early	23.7	Reading	21.6	Watch	25.9
Grow up	21.1	Hour	23.7	Studies	21.6	24 h	22.2
Night	21.1	Hurry	23.7	Train	21.6	Love	22.2
Past	21.1	Morning	23.7	College	19.6	Money	22.2
Seasons	21.1	Running	23.7	Wait	19.6	Not coming back	22.2
Growth	18.4	Sleeping	23.7	Lunch	17.6	Prayer	22.2
Punctuality	18.4	Week	23.7	Chatting	15.7	Writing	22.2
Schedule	18.4	24 h	21.1	Family	15.7	No future	18.5
Sleeping	18.4	Winter	21.1	Games	15.7	Library	18.5
Summer	18.4	Death	18.4	Home	15.7	Practice	18.5
Watch hand	18.4	Evening	18.4	Cell phone battery	15.7	Present	18.5
Working	18.4	Free time	18.4	Schooling	15.7	Significant	18.5
Death	15.8	Memorable	18.4	Time management	15.7	Achievement	14.8
Evening	15.8	Night	18.4	Aged	13.7	Aged	14.8
History	15.8	Slow	18.4	Future	13.7	Aim	14.8
Late	15.8	Working	18.4	Prayer	13.7	Happy	14.8
Short	15.8	Boring	15.8	Punctuality	13.7	Music	14.8
Slow	15.8	Present	15.8	Speed	13.7	Place	14.8
Sun	15.8	Seasons	15.8	Talking	13.7	Profit	14.8
Wait	15.8	Timetable	15.8	Breakfast	11.8	Reading	14.8
Wasted	15.8	Endless	13.2	Day	11.8	Relationship	14.8
Week	15.8	Forget	13.2	Dinner	11.8	Responsibility	14.8
Eternal	13.2	Long	13.2	Experimentation	11.8	Schooling	14.8
Hurry	13.2	Money	13.2	Happy	11.8	Second	14.8
Live	13.2	Autumn	10.5	24 h	11.8	Studies	14.8
Long	13.2	Century	10.5	Memorable	11.8	Thought	14.8
Morning	13.2	Midnight	10.5	Movies	11.8	Train	14.8
Noon	13.2	Past	10.5	Playing	11.8	Birth	11.1
Silence	13.2	Periods	10.5	Tea	11.8	Change	11.1
Time management	13.2	Rush	10.5	Teacher	11.8	Competitor	11.1
Calendar	10.5	Short	10.5	TV	11.8	Computer	11.1
Change	10.5	Spring	10.5	Wake up	11.8	Death	11.1
Childhood	10.5	Young	10.5			Distance	11.1
Duration	10.5	Wait	10.5			Eating	11.1
Fast	10.5					Eternal	11.1
Limited	10.5					Everything	11.1
Line	10.5					Exercise	11.1
Paper	10.5					Fast	11.1
Relax	10.5					Helping	11.1
Rise	10.5					Marriage	11.1
Time zone	10.5					Mind	11.1
Train	10.5					Cell phone battery	11.1
Winter	10.5					Necessary	11.1
						Past	11.1
						Personality	11.1
						Precious	11.1
						Problem	11.1
						Remember	11.1
						Research	11.1
						Temple	11.1
						Useful	11.1
						Victory	11.1
						Wake up	11.1
						Watch movie	11.1

#### How Well Do Categories of Associations Agree?

In different cultures, different specific words might be used to denote the same things. If that is so, there should be more agreement if one uses categories of words instead of the specific words themselves. To examine this possibility, the four authors independently clustered all terms shown in Table 1 (first translated into English, if necessary) in the German and Indian samples into clusters of words. If there was disagreement about a specific clustering, the issue was discussed until consensus was reached. Table 2 shows the resulting common and specific categories, illustrated by two sample associations each.

**Table 2:** Common and specific categories of associations with “time” across Indian and German respondents

Common categories	Specific Indian	Specific German
Time measurement devices (clock, watch)	Family and friends (marriage, friends)	Seasons (spring, winter)
Units of time measurement (second, minute)	Worship (prayer, temple)	
Steps/periods in life (birth, young)	Strongly positive aspects (healing, auspicious)	
Negative uses of time (waste, rush)	Strongly negative aspects (shock, suffer)	
Time perspectives (memory; future)	Time-dependent resources (cell phone battery, catch bus)	
Punctuality (late, wait)	Meals (breakfast, dinner)	
Passage of time (endless, running, fast)		
Subjective value of time (money, valuable)		
Uncomfortable aspects of time (stress, boring)		
Mundane activities (sleep, work, train)		
Student work (examination, working, class)		
Limitedness of time (limited, time management)		
Structuring time (schedule, timetable)		
Leisure (holiday, TV, music, book)		

To estimate the upper limit of agreement across cultures, we were quite liberal in judging whether a given category in one culture also existed in the other. For instance, there are a number of frequently associated expressions in the German samples that fit the common category “units of time measurement,” such as “second,” “minute,” and “hour,” but only one (“second”) in the Indian samples; yet we still postulated the existence of that category also in the Indian samples. Indeed, this kind of analysis reveals a stronger degree of agreement. Of the 21 categories we identified, 14 (67%) were common across cultures. For most categories we arrived at for the German samples we found fitting associations produced by the Indian samples. The only category specific to the German samples we could identify was “seasons,” which obviously do not exist in India in the form one encounters them in the northern hemisphere because of the country’s subtropical location. However, we could identify several specific categories of associations for the Indian samples. The Indian but not the German participants’ associations referred to family and friends, to worship-related issues, to strongly positive (e.g., “auspicious”) and negative (e.g., “shock”) issues, to time-dependent resources such as cell phone batteries, and to meals.

### Discussion

To examine the question of whether there is a universal semantic structure of time or not, we had culturally and linguistically quite diverse participants produce associations with the stimulus “time.” A high agreement among the different samples would lend credence to the universality assumption. However, the present analysis leaves us with mixed results. Based on the agreement of specific associative responses, our results indicate a common semantic structure in the two German samples across two languages (German and English), and still a substantial agreement for the two Indian samples (Kannada and English). However, the cross-cultural comparison revealed only modest agreement between the two samples that used the (common) English language and only a small amount of agreement for the two samples that used their mother tongues (Kannada and German). If instead of specific associations, categories of associations are used as the unit of analysis, the agreement among the two cultures is substantially higher, but this result may be seen as an upper limit. The categorical analysis also revealed some peculiarities that could be regarded as fitting the two cultural distinctions introduced above. For instance, only the Indian samples referred to family and friends, a topic especially important for people with P-time use; and the association of worship-related terms (e.g. “prayer”, or “eternal”) in the Indian samples might indicate a prevalent religious background that might be connected to a cyclical notion of time inherent in the Indian religious systems.

The present results seem to leave open the question if the categorical analysis was more indicative of the underlying semantic structure of time than the results for the specific associative responses, which could have been brought about by different word use. Thus, to further explore whether there might be a strong similarity among the semantic structures of time in the two cultures, participants should make judgments on a selection of terms that is representative for both cultures. This was done in Study 2.

## Study 2: Do Basic Semantic Dimensions and Categories of Time Differ across Cultures?

In Study 1, the results of the categorical analysis were somewhat contradictory to the results based on specific associations with “time.” The former left open the possibility that there might be a universal semantic structure of time after all, if potential differences in word use are accounted for. To examine this question further, and to explore underlying semantic dimensions of time more systematically, stimuli are needed that are as culturally unbiased as possible or, in other words, are representative of both the Indian and the German vocabulary. With such a representative list of time-related stimuli, the aim of Study 2 was to explore semantic dimensions of time as well as common clusters or categories across the four samples. Both objectives can be tackled by having participants make similarity judgments for pairs of time-related words and analyzing the resulting similarity matrix by the method of multidimensional scaling (see below).

### Methods

#### Participants

New samples of participants were recruited via local mailing lists and social media. The Kannada- and the English-speaking samples in India were collected from Karnatak University and Bangalore University and consisted of *n* = 23 (mean age = 21.7 years, 52% female) and *n* = 20 (mean age 22.2 years, 65% female) participants, respectively. German samples were collected at Chemnitz University of Technology, and *n* = 50 students (mean age, 23.1 years, 70% female) participated in the German-language condition and *n* = 37 (mean age = 25.8, 73% female) in the English-language condition. The mean self-ratings of participants’ English proficiency on a scale of 1 (*none*) to 10 (*fluent*) was 7.9 for the Indian and 6.9 for the German sample. Data were collected online, and participants could either obtain course credit or participate in a lottery in which they could win a book coupon.

#### Selection of Stimuli

Stimuli were selected from the association terms obtained in Study 1. All authors selected terms independently, following three criteria: Terms (i) should be frequent associations (see Table 1) and (ii) should cover as many categories as possible (see Table 2); and (iii) there should be a good balance of terms associated in the two cultures. To arrive at a suitable list, we also had to make a pragmatic decision about the maximum number of stimuli. Please note that the similarity between all stimuli had to be judged, which yields *n*(*n*-1)/2 comparisons. In several preliminary trials it was found that there should not be more than *n* = 27 stimuli (yielding 27*26/2 = 351 similarity judgments) in order to keep participants motivated. After several revisions, we agreed on the following list of time-related stimuli: *age*, *appointment*, *bus*, *change*, *clock*, *day*, *endless*, *exam*, *fast*, *friends*, *happy*, *home*, *important*, *late*, *life*, *memory*, *money*, *prayer*, *punctuality*, *running*, *sleep*, *stress*, *time*, *travel*, *TV*, *waiting*, and *work*.

#### Procedure

The order of presentation for the stimulus pairs was determined randomly and an online questionnaire using the program LimeSurvey was constructed, along with a second questionnaire with a reversed order of presentation. These two versions were prepared both in English and in the respective mother tongues (Kannada and German). In each country, each participant was randomly assigned to one of the two respective versions. Participants were asked to make judgments of association strength by typing in a number between 0 (*no association*) and 10 (*strong association*) for all stimulus pairs.

#### Analysis of Similarity Ratings

The resulting association or similarity matrix was analyzed using the SPSS program PROXSCAL, a prominent tool for performing multidimensional scaling analyses (e.g., Borg and Groenen 2005). Originally, multidimensional scaling (MDS) was developed to reveal dimensions or latent factors underlying any kinds of objects by relying on judgments of their similarity or distance. The technique has since increasingly been used to visualize similarities between stimuli and thus arrive at conclusions about clusters or categories for these stimuli. In our study, we were interested in both kinds of results. MDS can be applied to any similarity matrix. But as the matrix for a given participant might exhibit highly idiosyncratic associations, we always used the mean associations calculated over participants in each of the four samples. Following the recommendations of Borg et al. (2010), we used multiple random starts (*n* = 1000) for the initial configuration and a stress convergence of .00001 in all analyses. Mostly for pragmatic reasons, we concentrated on two-dimensional MDS solutions.^7^

### Results

**Fig. 1:**
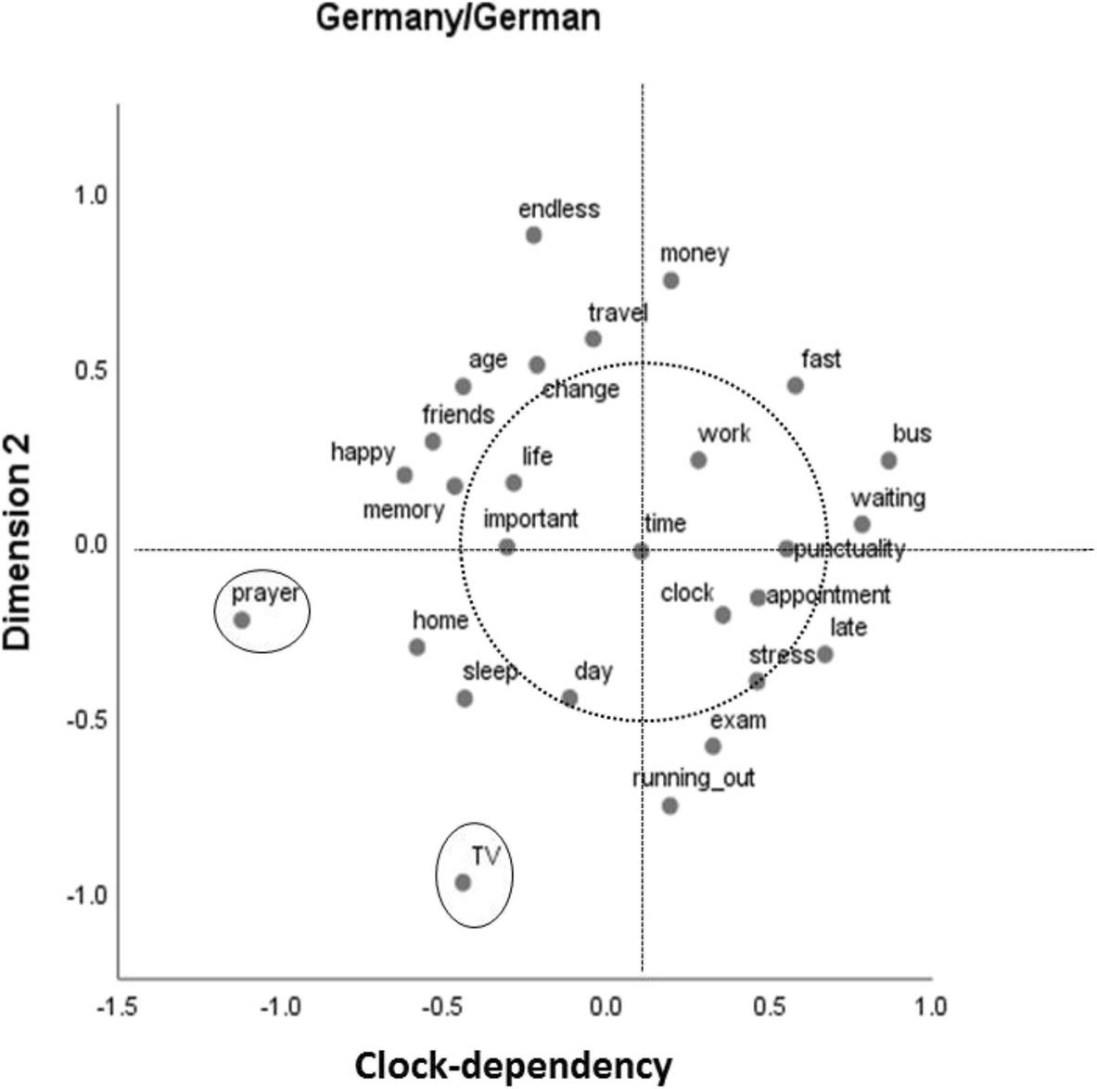
Two-dimensional multidimensional scaling solution for the German sample that used German stimuli (normalized stress = .08). The dotted circle surrounds central categories around “time,” and the ellipses mark two outliers

The two-dimensional solution of the MDS analysis for the German sample that judged German stimuli can be seen in Fig. 1. As could be expected, the stimulus “time” is accorded a central position in the figure. To help the dimensional analysis, we always added horizontal and vertical lines at this position, as well as a circle around the stimulus “time,” with a radius of 0.5 scale units. The horizontal dimension can be interpreted as “clock dependency.” If one looks at the stimuli to the right of “time,” one finds that most of the terms are strongly dependent on time units, as measured by clocks. Time measurement does not play a central role for most of the stimuli found to the left of the term “time.” The vertical dimension is much harder to make sense of.

The configuration of stimuli in Fig. 1 does not suggest highly segregated categories but two stimuli stand out immediately: “TV” and “prayer” (left lower quadrant). These two stimuli stem from the exclusively Indian categories in Table 2. A look around the stimulus “time” reveals that “clock” and “work” are most central, followed by “appointment,” “punctuality,” “important,” “life,” “day,” and “stress.” So this collection of stimuli (within the circle in Fig. 1) can be seen as describing the core of German students’ semantic structure of time.

**Fig. 2:**
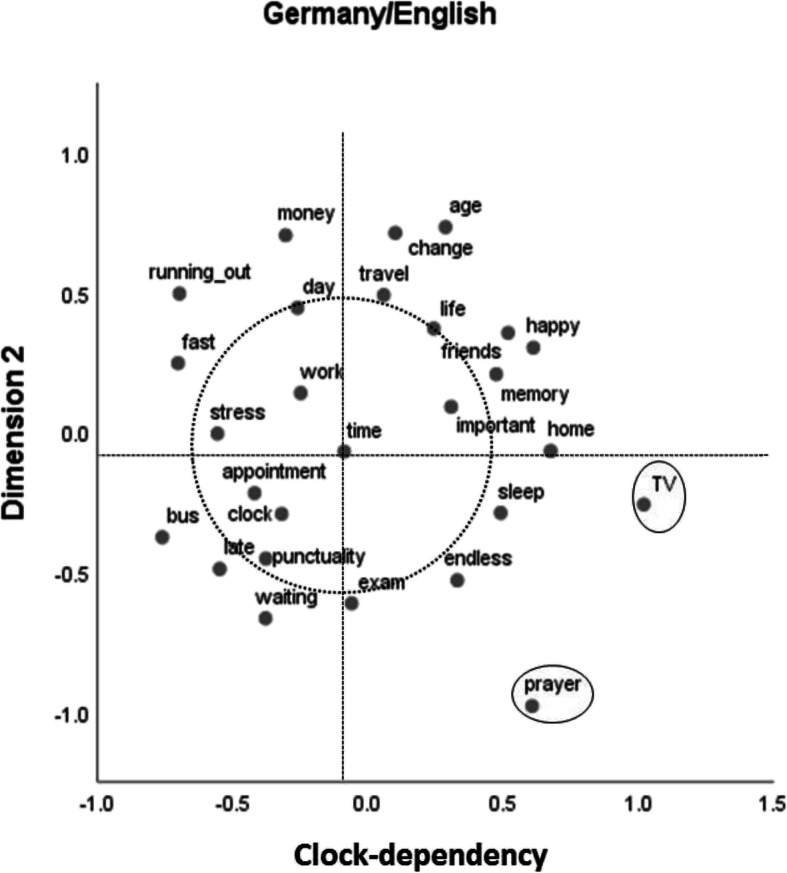
Two-dimensional multidimensional scaling solution for the German sample that used English stimuli (normalized stress = .08). The dotted circle surrounds central categories around “time,” and the ellipses mark two outliers

Figure 2 shows the two-dimensional MDS solution for the German sample with English stimuli. It is more or less a mirror image (in respect to the *x* axis) of the results depicted in Fig. 1. Again, the horizontal dimension (now reversed in comparison to Fig. 1) can be described as clock dependency. As for the results in Fig. 1, no plausible description could be found for the vertical dimension, for which also the ordering of stimuli partly differs from the results depicted in Fig. 1. A prominent example is the position of “running out” (German: ablaufen), which in German also has the connotation of ending abruptly.

Again, “TV” and “prayer” stand out, and the core stimuli that can be found in close proximity to the central stimulus “time” (see circle around it) are identical to those identified for the sample with German stimuli: “work,” “clock,” “appointment,” “punctuality,” “stress,” “important,” “day,” and “life.”

**Fig. 3:**
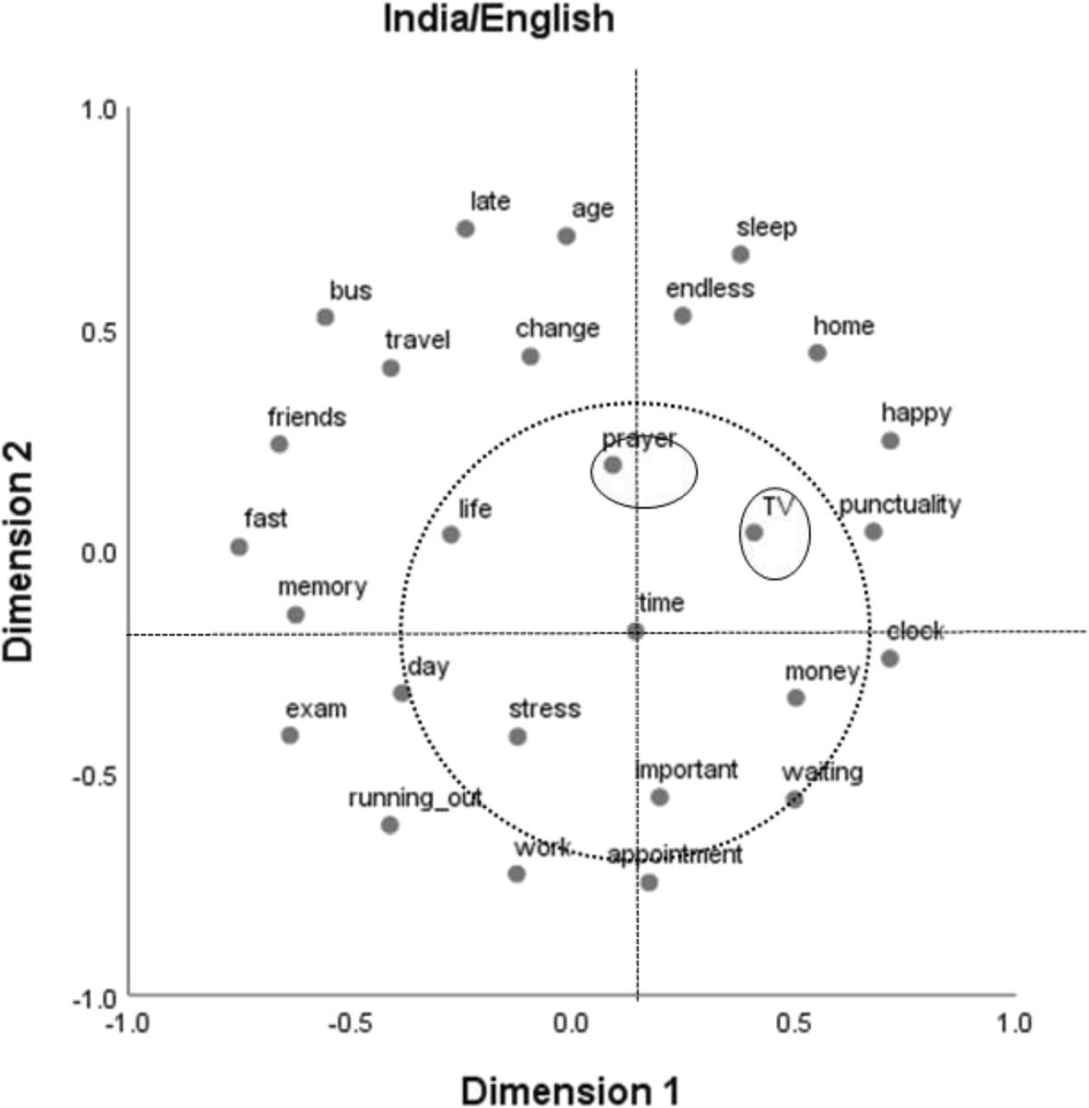
Two-dimensional multidimensional scaling solution for the Indian sample that used English stimuli (normalized stress = .15). The dotted circle surrounds central categories around “time,” and the ellipses mark terms that were outliers in the German samples

The results for the Indian sample that used English stimuli can be found in Fig. 3. The two dimensions offered by the MDS solution are hard to interpret. It seems that the ordering of stimuli in the figure does not follow any plausible dimension. Interestingly, now the two stimuli that could be regarded as outliers in the German samples—“TV” and “prayer”—seem to be central to the notion of “time,”^8^ along with “stress,” “life,” “important,” “money,” “waiting,” and “day.”

**Fig. 4:**
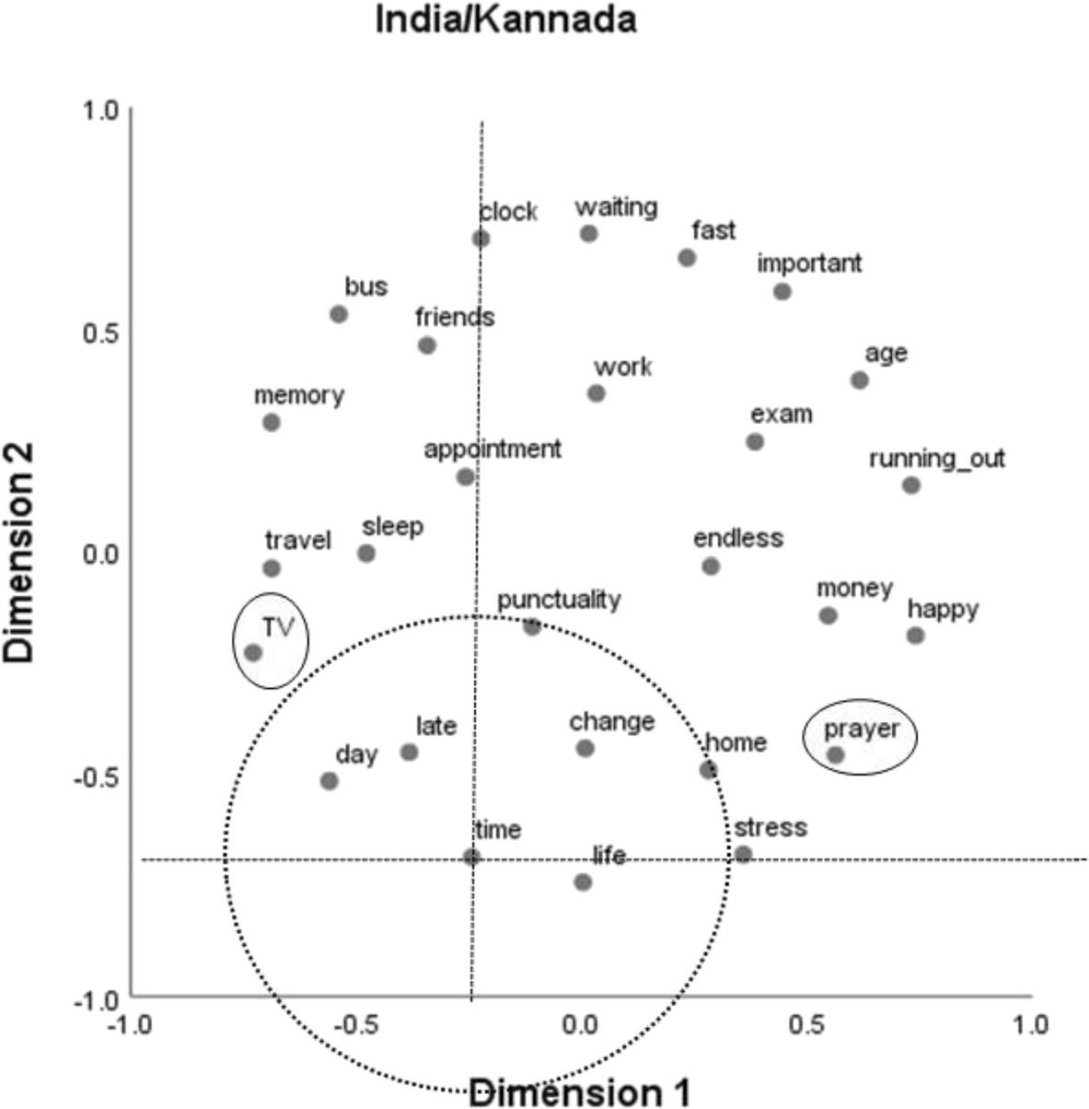
Two-dimensional multidimensional scaling solution for the Indian sample that used Kannada stimuli (normalized stress = .15). The dotted circle surrounds central categories around “time,” and the ellipses mark terms that were outliers in the German samples

Finally, Fig. 4 shows the results for the Indian sample working with Kannada stimuli. As in the other Indian sample, no clear interpretation could be found for the two dimensions identified by the MDS analysis. In contrast to the results for all other samples, “time” does not occupy a central position. However, as in the other Indian sample, “TV” and “prayer” do not stand out in any way. The stimuli most central to the notion of “time” are “life,” “late,” “day,” “change,” “punctuality,” and “home.” Only two of these, “life” and “day,” are also found central in the solution for the Indian sample with English stimuli (Fig. 3).

### Discussion

The aim of Study 2 was to find out whether common semantic dimensions of time as well as common clusters or categories could be identified across the two cultures or whether differences prevail. Overall, our results speak for the latter. But there are some qualifications. Only one dimension could be plausibly interpreted at all, and only in the two German samples. This dimension, which we termed clock dependency, differentiates between stimuli that are strongly connected to the measurement of time versus those that are not. Apparently, German students’ semantic structure of time is very much ordered along this dimension, whereas for the Indian students, such a dimension does not seem to exist. The stimulus “bus” might be a good candidate stimulus to illustrate the difference between the two cultural groups. In the German samples, it can be found at the extreme end of the clock dependency dimension, in close proximity to stimuli such as “fast,” “stress,” or “punctuality” (Figs. 1 and 2), whereas in the Indian samples, close neighbors to “bus” are “friends” and “travel” (Figs. 3 and 4). This difference might well be connected to the bus systems in the two countries. Whereas in Germany, buses are usually quite punctual and do not wait for late passengers, in India, bus schedules are usually quite “flexible” and buses often run frequently and at almost all times of the day.

Commonalities across the two cultures should be most easily identified for the two samples that used English stimuli. If one looks at the respective core stimuli around “time” (within the circles in Figs. 2 and 3), the amount of agreement is 50% (4 of 8). This is substantially lower than the amount of agreement across the two German samples (100%, that is, 8 of 8). This cross-cultural difference is also exemplified in the relative positions of the stimuli “TV” and “prayer.” These stimuli are well integrated in both Indian MDS solutions but are outliers in the German results. Whereas “TV” might be seen as an indicator of P-time use, “prayer” could be seen as indicating Indian students being concerned with their karma, as would be expected if the cyclical notion of time (the second variety mentioned above) was relevant for them.

Apart from genuine cross-cultural differences (not necessarily connected to language although we used language to measure them) language itself also seems to have made a noticeable difference in Study 2, especially concerning the Indian samples. For the German samples, there are some noticeable differences only in the vertical MDS dimension (Dimension 2), but the two MDS solutions for the Indian samples are much further apart. The amount of agreement on the central concepts for the English and the Kannada samples is only 33% (2 of 6), even smaller than that for the two English samples across the two cultures. Whereas the difference between the German samples may be due to different connotations of the respective German expressions and their English translations, such a difference in connotation cannot account for the strong difference between the Indian samples. For instance, when Indians dealt with English expressions, time was treated as important and strongly associated with money (Fig. 3), which was not the case when Indians made their judgments using Kannada expressions (Fig. 4). The use of the mother tongue might have facilitated the occurrence of less systematic (and maybe more emotional—see Table 1) associations than when using a foreign language, which would be consistent with results from decision-making research (Keysar et al. 2012). Such a differential effect might be the more pronounced the more distant the two languages in question are. Whereas German and English are members of the same Indo-European family of languages, Dravidian languages such as Kannada differ in many respects from Indo-European languages (Sjoberg 2009; Steever 1987). Moreover, the association structure in Fig. 4 might also indicate that the terms we chose might not have been representative for that sample, after all: There, “time” occupies only a peripheral position.

### General Discussion

Is there a universal semantic structure of time? The results of our two studies do not rule out that there might be a universal common core but they strongly indicate marked cross-cultural differences in how people think about time. These differences seem to be further accentuated by the use of specific languages. The categorical analysis in Study 1 left open the possibility that the low cross-cultural agreement found for the original associations to the stimulus “time” might have been an underestimation. However, the results in Study 2, with a careful selection of time-related stimuli, do not support such a view.

In Study 2, we could identify only one meaningful interpretation of the dimensions produced by MDS, and this was only possible for the German samples. One major dimension of German students’ implicit structure of time obviously represents what we termed clock dependency. For them, it seems to make a strong difference how important the measurement of time is for a respective activity, situation, or judgment. It is, for instance, very important to be aware of the measurement of time when having an appointment, wanting to be punctual, being in a waiting situation, or intending to catch a bus, whereas this awareness of exact timing is less important when at home, sleeping, or with friends. For the Indian counterparts no such interpretation could be found. This difference can be well connected to the distinction between monochronic and polychronic time use. In an M-time culture such as that in Germany, in which people tend to concentrate on one thing at a time, and in which keeping schedules is very important, such a semantic structure component of time makes perfect sense. Clock dependency would not, however, be expected in P-time cultures, such as that in India, in which completing human transactions is more important than meeting deadlines or being on time.

The cross-cultural difference in respect to clock dependency may also be indicative of the second potentially relevant cultural difference introduced above: that between a linear and a cyclical notion of time. A consequence of believing in the latter would be that there are further chances in future lives to be happy, to have friends, or to lead a good life, whereas in cultures adhering to the former, there is only one chance to obtain these positive aspects of life. Indeed, in the MDS solutions for the German samples, the stimuli “happy” and “friends” as well as “life” and “memory” are found in close proximity (see Figs. 1 and 2), whereas in the solutions for the Indian samples, these stimuli are in some cases quite far apart (see Figs. 3 and 4). As already mentioned, a second indication for a potential impact of the belief in future lives might be the position of the associative response “prayer.” This associative response was produced only in the Indian samples, and it also occupies a central role (Fig. 3) or at least is not an outlier (Fig. 4) in the MDS solutions for these samples, in contrast to the results for the German samples (Figs. 1 and 2).

There are strong indications that the perception and use of time as well as judgments about time may be quite different across different cultures (e.g., Block et al. 1996; Brodowsky et al. 2008; Levine 1997; Levine et al. 1980; Unger et al. 2014), but these differences do not necessarily indicate that there are also cross-cultural differences in the semantic structure of time. Our results suggest that these observable differences in respect to dealing with time-related issues may be fundamentally rooted in different semantic structures of time. However, for people speaking several different languages, several overlapping structures of time might exist, especially if these languages differ strongly. These different semantic structures of time might be differentially activated depending on the language used.

In our studies, we operationalized semantic structure by relying on participants’ associative responses. Doing this does not necessitate a distinction between objective and subjective time as made, for instance, in the phenomenological tradition (e.g. Cornejo and Olivares 2015). Instead, the assumption of a universal semantic structure of time would just implicate a strong similarity between the (subjective) associations to time people have across different cultures. The method we used can be quite powerful but it makes sense to complement it with a qualitative approach in future studies, to find out more about how people think about time.

## Footnotes

1 Saint Augustine. (n.d.). BrainyQuote.com. Retrieved January 18, 2017, from BrainyQuote.com: https://www.brainyquote.com/quotes/quotes/s/saintaugus108119.html

2 The difference in cultures underlying the monochronic–polychronic distinction is largely that between low-context and high-context cultures, which is characterized by the way in which information is transmitted. In high-context cultures, much information is preprogrammed in the receiver and the setting with only minimal information transmitted in the message itself, whereas in low-context cultures it is the reverse (Hall 1976, p. 101).

3 Whereas after death, in Hinduism, the soul (e.g., atman) just moves from one human body to another, in Buddhism, there is no soul that could be reincarnated in another body—thus, only the individual’s karmic tendencies continue in a new body.

4 This discrepancy in number of associations is proportional to the respective sample sizes. It should not, however, have a systematic impact on our results because our analysis does not depend on absolute but on relative numbers (see below).

5 An example: In the Indian sample that used English, 47 associative responses were produced by more than 10% of the participants. The respective number for the Indian sample that used Kannada was 63. So, for each of the associative responses of the former group (smaller number of associations), we looked at whether the association also appeared in the latter group and found 24 associations shared by both samples.

6 The percentages of agreement might be seen as conservative figures because some associations are not identical verbatim but still quite similar (e.g., “endless” and “eternal”, or “watch” and “watch hand”).

7 In all four samples, the stress measure was substantially reduced for the two-dimensional compared to the one-dimensional solution, and there was little additional reduction by adding one more dimension. Moreover, two-dimensional solutions are easier to interpret than three-dimensional ones.

8 One possible explanation for the centrality of TV might be that in India, the TV can be used as a clock because the time of the day is always visible on TV screens.
